# Associations between Sarcopenia and trajectories of activities of daily living disability: a nationwide longitudinal study of middle-aged and older adults in China from 2011 to 2018

**DOI:** 10.1186/s13690-024-01329-x

**Published:** 2024-06-25

**Authors:** Lei Lan, ShiMiao Shao, Xiaowei Zheng

**Affiliations:** 1 State Key Laboratory for Diagnosis and Treatment of Infectious Diseases, National Clinical Research Center for Infectious Diseases, Collaborative Innovation Center for Diagnosis and Treatment of Infectious Diseases, The First Affiliated Hospital, School of Medicine, Zhejiang University, 79 Qingchun Road, Hangzhou, Zhejiang Province 310003 China; 2 Public Health Research Center, Department of Public Health and Preventive Medicine, Wuxi School of Medicine, Jiangnan University, 1800 Lihu Road, Binhu District, Wuxi, Jiangsu Province 214122 China

**Keywords:** Sarcopenia, Disability, Activities of daily living, Trajectory, Obesity

## Abstract

**Background:**

Sarcopenia is an age-related clinical syndrome, which is associated with numerous adverse outcomes among older adults. The relationship between sarcopenia and activities of daily living (ADL) disability has been studied in China, but these findings usually focused on a single time point. The patterns of ADL can change over time and vary among individuals. Therefore, it is necessary to explore the association between sarcopenia and trajectories of ADL disability.

**Methods:**

According to Asian Working Group for Sarcopenia (AWGS) 2019 criteria, muscle mass, muscle strength, and physical performance measurements were measured to diagnose sarcopenia. A six-item ADL score was used to measure ADL disability, and trajectories of ADL disability were identified by the latent class trajectory modelling (LCTM). Multiple logistic regression models were performed to examine the association between sarcopenia and trajectories of ADL disability.

**Results:**

Among 9113 middle-aged and older adults, three trajectories of ADL disability were determined according to changes in ADL score during follow-up, including a mild-high trajectory (*n* = 648, 7.11%), followed by the low-mild trajectory (*n* = 3120, 34.24%) and low-low trajectory (*n* = 5345, 58.65%). After adjustment for covariates, severe sarcopenia was significantly associated with higher risks of being in the mild-high trajectory group (OR = 3.31, 95%CI: 2.10–5.22) and the low-mild trajectory group (OR = 1.44, 95%CI: 1.05–1.98), compared with the low-low trajectory group. This association was still observed when stratified by age and gender. In addition, participants with sarcopenic obesity were associated with a higher risk of ADL disability (OR = 3.99; 95% CI: 2.50–6.09).

**Conclusions:**

Among the middle-aged and older Chinese adults, sarcopenia and sarcopenic obesity were both associated with persistent higher trajectories of ADL disability. It suggested that early interventions to sarcopenia and sarcopenic obesity among the middle-aged and older adults may reduce the progression of ADL disability.

**Supplementary Information:**

The online version contains supplementary material available at 10.1186/s13690-024-01329-x.

**Table:** 

**Text box 1. Contributions to the literature**
• Evidence on the relationship between sarcopenia and ADL disability trajectories is limited in China.
• Sarcopenia and sarcopenic obesity were associated with persistently higher ADL disability trajectories.
• Early recognition and management of sarcopenia among the middle-aged and older adults in China may reduce the progression of ADL disability.

## Background

The activities of daily living (ADL) is considered the basic skills of caring for oneself independently, such as eating or bathing, and ADL disability is defined as partly or entirely unable to perform basic ADL, which may lead to the poor quality of life in older adults [1, 2]. With a rapidly aging population, China has a heavy burden of ADL disability [3–5]. A recent study in China reported that older adults with ADL disability is projected to increase to 96.2 million in 2060, which brings a significant economic burden on individuals and society [6]. Therefore, identification of potential risk factors of ADL disability and development of effective preventive strategies are necessary.

Sarcopenia is an age-related and progressive skeletal muscle disease characterized by reduction of muscle strength and function, which is prevalent in older adults [7–9]. It is reported that the estimated prevalence of sarcopenia was ranging from 5.5 to 25.7% among older Asian people [10]. Previous studies have shown that sarcopenia in older adults was independently associated with disability and poor physical function [11–14].In addition, compared with sarcopenia and obesity alone, sarcopenia obesity (SO) was associated with an increased risk of ADL disability [15–18]. Although the relationship between sarcopenia and ADL disability have been verified by linear mixed effects models in longitudinal studies [12, 19, 20], it might result from incomplete data and fail to precisely capture the dynamic change of ADL disability. To date, the latent class trajectory modelling (LCTM) has been increasingly recognized for its usefulness in characterizing dynamic change and identifying unobserved heterogeneity in trajectories among individuals [21–23]. However, few studies have focused on the association between sarcopenia and trajectories of ADL disability in China. Hence, in this study, we used the LCTM to determine trajectories of ADL disability based on repeated measures of ADL score data from China Health and Retirement Longitudinal Study (CHARLS). We aim to evaluate the role of sarcopenia and SO in the temporal changing pattern of ADL disability.

## Methods

### Study population

As a representative survey of middle-aged and older adults in China, CHARLS is an ongoing nationwide cohort study that uses a multistage clustering sample method to select participants [24]. A total of 17,708 participants recruited from 28 provinces within China were included at baseline (2011–2012, Wave 1). CHARLS respondents were followed up every 2 years, using a face-to-face computer-assisted personal interview. Three subsequent follow-ups were carried out among survivors in 2013–2014 (Wave 2), in 2015–2016 (Wave 3) and 2017–2018 (Wave 4), respectively. The details of the CHARLS data are available at its website (http://charls.pku.edu.cn/en).

**Fig. 1:**
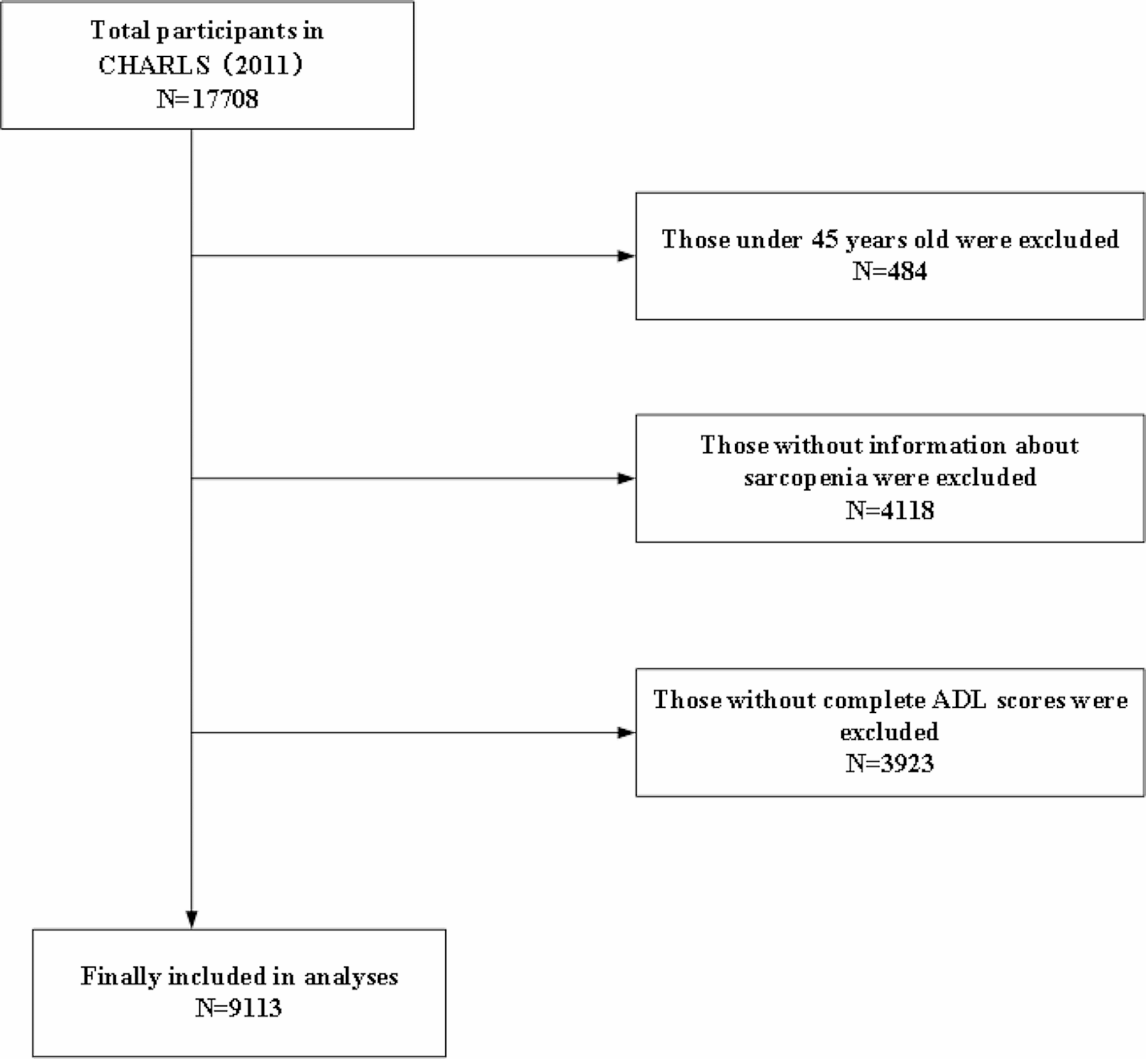
Flow chart of sample selection and the exclusion criteria in this study. CHARLS, China Health and Retirement Longitudinal Study; ADL, activities of daily living

In this analysis, the inclusion criteria were as follows: aged ≥ 45 years; reported the information about sarcopenia in baseline; completed four follow-ups (Wave 1 to Wave 4). Finally, a total of 9113 out of 17,708 respondents were eligible for subsequent analysis. More details regarding eligibility and selection of participants are shown in Fig. 1.

### Measurements of ADL

In CHARLS, ADL was measured by using the six-item scale, including dressing, bathing, eating, transferring, toileting, and urination controlling [25]. Scores ranging from 0 to 4 were assigned to four response options for each item (1. no difficulty; 2. difficult but achievable; 3. some difficulties and need help; 4. unable to complete). Participants were defined as having the ADL disability if they lacked complete independence in any item. The ADL scores were calculated for each participant in every survey period, with higher score indicating more severe ADL disability.

### Measurements of sarcopenia

According to the Asian Working Group for Sarcopenia (AWGS) 2019 criteria [10], sarcopenia was defined as age-related loss of muscle mass, plus low muscle strength, and/or low physical performance. Applying the bioimpedance analysis, muscle mass was measured as appendicular skeletal muscle mass (ASM). Then, the height-adjusted muscle mass was calculated by dividing the ASM by the square of the height in meters (ASM/Height^2^). Based on a previous study [26], the ASM in the Chinese population was estimated using the following reported physical measurement equation:

ASM = 0.193×weight(kg) + 0.107×height(cm)-4.157×gender-0.037×age(years)-2.631.

(Weight in kg; height in m; age in years; gender: 1, for men and 2, for women).

Similar to previous studies [27, 28], low muscle mass was defined as height-adjusted muscle mass of lower than 7.01 kg/m^2^ in men and lower than 5.27 kg/m^2^ in women. As recommended by AWGS 2019 [10], low muscle strength was defined by grip strength < 28 kg for men and < 18 kg for women, respectively. Meanwhile, low physical performance was defined as 6-m walk < 1.0 m/s, or 5-time chair stand test ≥ 12 s, or Short Physical Performance Battery (SPPB) score < 9 [10].

Based on the presence of *a*. low muscle mass, *b*. low muscle strength, and *c*. low physical performance, the sarcopenia outcomes were defined as non-sarcopenia (absence of *a*, *b*, and *c*), possible sarcopenia (presence of *b* with or without *c*), sarcopenia (presence of *a* and *b*/*c*) and severe sarcopenia (presence of *a*, *b*, and *c*). Moreover, according to the World Health Organization(WHO) adult classification, obesity was defined as a body mass index (BMI) of ≥ 30.0 kg/m^2^ and overweight as a BMI of ≥ 25.0 kg/m^2^. We used the combination of the diagnostic criteria of sarcopenia and obesity to define sarcopenic obesity (SO). Details of operational and equipment information for measurements are available in the overview of the CHARLS study [24].

### Covariate assessments

For the CHARLS 2011 survey, the demographic information, including age, sex, living place (urban vs. rural), educational level (below primary school, primary school, middle school, and high school or above), medical history (hypertension, dyslipidemia, diabetes mellitus), smoking status (past smoking vs. never smoking), drinking status (past drinking vs. never drinking), blood glucose, BMI (the weight in kilograms divided by the square of the height in meters), systolic blood pressure (mm Hg), and diastolic blood pressure (mm Hg), were collected at baseline. Blood pressure was measured with an electronic sphygmomanometer (Omron HEM-7200 Monitor) after 5 min of rest in the sitting position and the average of three separate measurements was used. Hypertension was defined as systolic blood pressure ≥ 140 mm Hg, diastolic blood pressure ≥ 90 mm Hg, current use of antihypertensive medications, or self-reported history of hypertension. Dyslipidemia was defined as triglycerides ≥ 150 mg/dL, total cholesterol ≥ 240 mg/dL, high-density lipoprotein cholesterol < 40 mg/dL, low-density lipoproteins cholesterol ≥ 160 mg/dL, current use of the lipid-lowering medications, or self-reported history of dyslipidemia. Diabetes mellitus was defined as fasting glucose ≥ 126 mg/dL, glycosylated hemoglobin (HbA1c) ≥ 6.5%, treatment for diabetes mellitus, or self-reported history of diabetes. In addition, smoking history was categorized as “past smoking” or “never smoking,” and drinking history was categorized as “past drinking” or “never drinking” according to self-reported history of the participants.

### Statistical analysis

The latent class trajectory modelling (LCTM) approach was conducted using the TRAJ plugin procedure in SAS software, version 9.4 (SAS Institute, Inc) to generate distinct trajectories of ADL disability. The LCTM estimated the model parameters through the maximum likelihood method and assigned each participant to a trajectory with the greatest posterior probability. Four times points (Wave 1 to Wave 4) were used as the timescales. Besides, the optimal trajectories and shapes were chosen based on the Bayesian information criterion (BIC) and the interpretability of the trajectories. Besides, participants’ baseline characteristics were described as percentages for categorical variables, as means with standard deviation for normally distributed continuous variables and as medians with interquartile range for non-normally distributed continuous variables., the demographic and clinical characteristics were compared among individuals with different trajectories of ADL disability using ANOVA or Kruskal-Wallis test for continuous variables and χ^2^ test for categorical variables. After adjusting for the covariates, multinomial logistic regression models were computed to evaluate the association of sarcopenia and SO with trajectories of ADL disability. Meanwhile, the ‘low-low’ trajectory was set as the reference group in the logistic regression models. Additionally, according to sex and age, subgroup analyses were performed to estimate the association between the risk of sarcopenia and ADL disability trajectories. To evaluate the robustness of study results, we conducted a sensitivity analysis that included information of sarcopenic status and sarcopenic overweight status with a different grouping strategy. Participants were categorized as no sarcopenia, possible sarcopenia and sarcopenia, where the sarcopenia group included the sarcopenia group and the severe sarcopenia group in previous analyses. A two tailed *P* < 0.05 was considered to be statistically significant. All statistical analyses were conducted using SAS statistical software (version 9.4, Cary, NC).

## Results

### Estimated ADL disability trajectories

**Fig. 2:**
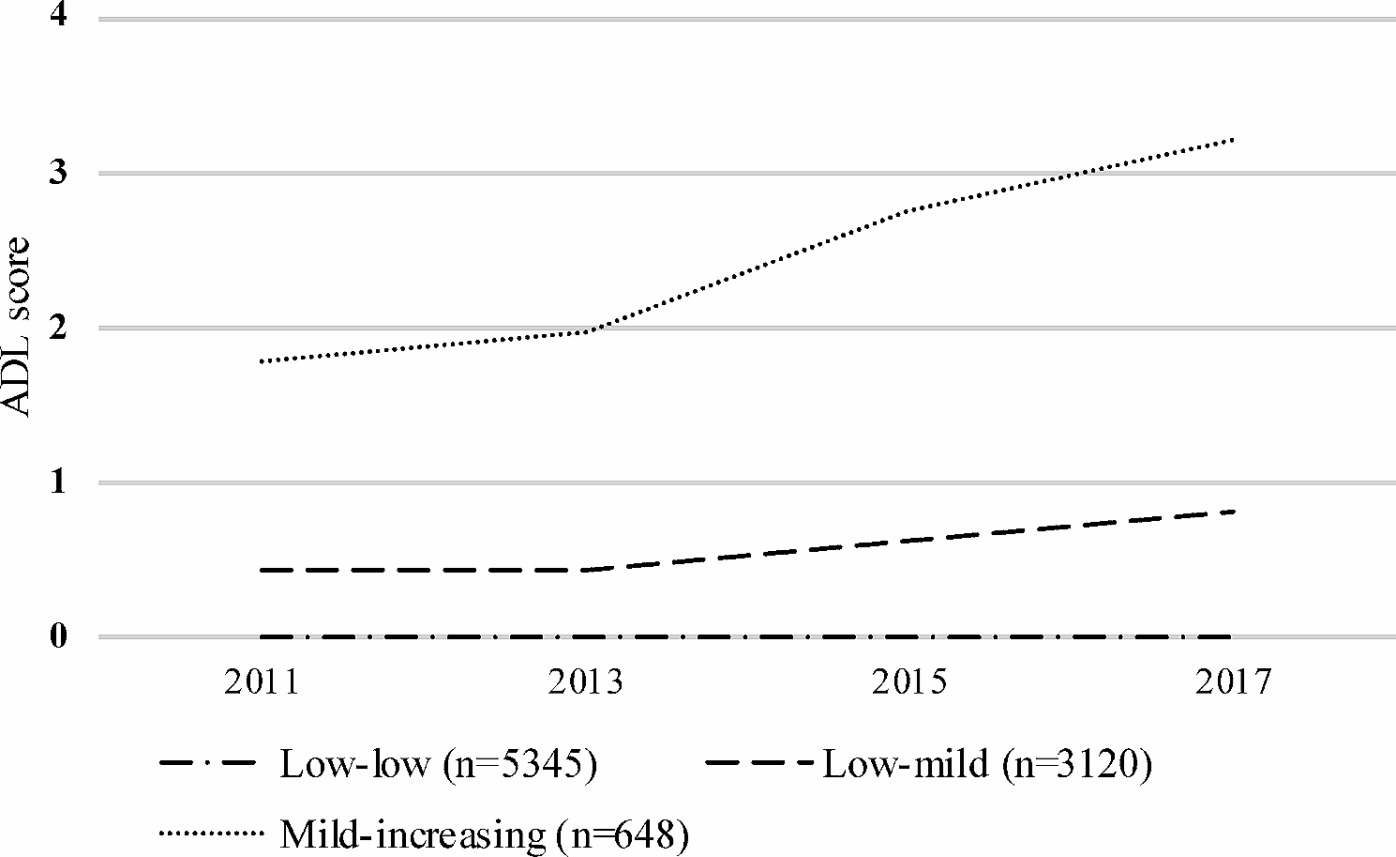
Trajectories of ADL among the participants of CHARLS in China from 2011 through 2018. ADL, activities of daily living; Four rounds of ADL measurements were obtained at Wave 1 to Wave 4(from 2011 through 2018), and were used to fit trajectories

Based on the model selection criteria, three distinct ADL disability trajectories throughout the follow-up were identified in the final model, namely, the mild-high trajectory (*n* = 648, 7.11%), the low-mild trajectory (*n* = 3120, 34.24%), and the low-low trajectory (*n* = 5345, 58.65%). The average posterior probabilities were 0.837, 0.900 and 0.846, respectively. Figure 2 shows three longitudinal patterns of ADL disability trajectories, plotted by survey time.

### Baseline characteristics of participants by ADL disability trajectories

In the present study, 9113 participants (4228 men and 4885 women) were included in the analysis, and the mean age was 58.46 ± 8.74 years. The baseline characteristics of the participants in each trajectory group of ADL disability are presented in Table 1. Among the three trajectory groups, there were significantly differences in age, sex, living place, education level, dyslipidemia, diabetes mellitus, smoking, drinking, blood glucose, systolic blood pressure and diastolic blood pressure (*P* < 0.05).

**Table 1:** Baseline characteristics of the participants of CHARLS in China from 2011 through 2012

Characteristics	ADL trajectories during follow-up			*P* value
	Low-low	Low-mild	Mild-high	
No. of subjects	5345	3120	648	
Age, years	56.62 ± 8.06	60.35 ± 8.80	64.46 ± 9.21	< 0.001
Sex, n (%)				
Male	2781(52.03)	1220(39.10)	227(35.03)	< 0.001
Female	2564(47.97)	1900(60.90)	421(64.97)	
Living place, n (%)				
Urban	1972(36.89)	910(29.17)	149(22.99)	< 0.001
Rural	3373(63.11)	2210(70.83)	499(77.01)	
Education level, n (%)				
Below primary school	1183(22.12)	1099(35.22)	304(46.91)	< 0.001
Primary school	2114(39.55)	1373(44.01)	260(40.12)	
Middle school	1332(24.92)	448(14.36)	64(9.88)	
High school or above	716(13.40)	200(6.41)	20(3.09)	
Medical history				
Hypertension, n (%)	1155(21.61)	664(21.28)	155(23.92)	0.479
Dyslipidemia, n (%)	434(8.12)	316(10.13)	83(12.81)	< 0.001
Diabetes mellitus, n (%)	242(4.53)	239(7.66)	63(9.72)	< 0.001
Smoking, n (%)	2217(41.48)	1104(35.38)	216(33.33)	< 0.001
Drinking, n (%)	2214(41.42)	1102(35.32)	208(32.10)	< 0.001
Blood glucose, mg/dl	108.19 ± 31.45	111.21 ± 38.25	112.94 ± 39.74	< 0.001
BMI, kg/m^2^	23.13(20.96–25.66)	23.16(20.74–25.99)	23.22(20.88–26.53)	0.083
SBP, mmHg	128.35 ± 19.17	130.53 ± 20.66	134.38 ± 22.26	< 0.001
DBP, mmHg	75.83 ± 11.56	75.08 ± 11.37	75.528 ± 11.72	< 0.001

BMI: body mass index; SBP: systolic blood pressure; DBP: diastolic blood pressure; Continuous variables are expressed as mean ± standard deviation, or as median (interquartile range). Categorical variables are expressed as frequency (percent)

### Association between sarcopenia status and ADL disability trajectories

In the multinomial logistic regression model with covariates adjusted, compared with participants with no sarcopenia, those with possible sarcopenia, those with sarcopenia, and those with severe sarcopenia had significantly increased risks of being in the low-mild trajectory group (OR = 1.53, 95%CI: 1.36–1.72 for those with possible sarcopenia; OR = 1.53, 95%CI: 1.28–1.83 for those with sarcopenia; OR = 1.44, 95%CI: 1.05–1.98 for those with severe sarcopenia) and the mild-high trajectory group (OR = 2.36, 95%CI: 1.89–2.96 for those with possible sarcopenia; OR = 2.47, 95%CI: 1.81–3.37 for those with sarcopenia; OR = 3.31, 95%CI: 2.10–5.22 for those with severe sarcopenia). Detailed results are presented in Table 2.

**Table 2:** Association between sarcopenia status and ADL trajectories among the participants of CHARLS in China from 2011 through 2018

Characteristics	Low-mild V.S. Low-low				Mild-high V.S. Low-low			
	Crude model OR (95% CI)	*P*	Adjusted model OR (95% CI)	*P*	Crude model OR (95% CI)	*P*	Adjusted model OR (95% CI)	*P*
**Sarcopenia status**								
No sarcopenia	1.00 (ref)		1.00 (ref)		1.00 (ref)		1.00 (ref)	
Possible sarcopenia	1.95(1.75–2.17)	< 0.001	1.53(1.36–1.72)	< 0.001	3.49(2.86–4.25)	< 0.001	2.36-1.89-2.96	< 0.001
Sarcopenia	2.46(2.11–2.87)	< 0.001	1.53(1.28–1.83)	< 0.001	2.90(2.23–3.78)	< 0.001	2.47(1.81–3.37)	< 0.001
Severe Sarcopenia	2.96(2.21–3.97)	< 0.001	1.44(1.05–1.98)	0.023	4.18(2.79–6.27)	< 0.001	3.31(2.10–5.22)	< 0.001

* Multivariable-adjusted for age, sex, living place, education level, smoking, drinking, body mass index, blood glucose, systolic blood pressure, antihypertensive medication, antidiabetic medication and medical history (dyslipidemia, diabetes, cancer, chronic lung disease, kidney disease, liver disease, arthritis, digestive disease, asthma)

Additionally, to explore the relationship between sarcopenia, obesity and ADL disability trajectories, the individuals without sarcopenia and obesity were set up as the control group. The results of the logistic regression suggested that, compared with individuals without sarcopenia and obesity, individuals with obesity had a significantly increased risk of being in the low-mild trajectory group (OR = 1.51, 95%CI: 1.36–1.68) and the mild-high trajectory group (OR = 2.26, 95%CI: 1.83–2.79). Furthermore, participants with SO had a higher risk of being in the low-mild trajectory group (OR = 1.82, 95%CI: 1.38–2.40) and the mild-high trajectory group (OR = 3.99, 95%CI: 2.50–6.09). Detailed results are presented in Table 3.

**Table 3:** Association between sarcopenic obesity and ADL trajectories among the participants of CHARLS in China from 2011 through 2018

Characteristics	Low-mild V.S. Low-low				Mild-high V.S. Low-low			
	Crude model OR (95% CI)	*P*	Adjusted model OR (95% CI)	*P*	Crude model OR (95% CI)	*P*	Adjusted model OR (95% CI)	*P*
**Sarcopenic obesity status**								
Control	1.00 (ref)		1.00 (ref)		1.00 (ref)		1.00 (ref)	
Sarcopenia only	1.24(1.05–1.48)	0.012	1.13(0.90–1.41)	0.297	1.38(0.92–2.06)	0.122	0.99(0.59–1.66)	0.973
Obesity only	2.11(1.91–2.33)	< 0.001	1.51(1.36–1.68)	< 0.001	2.87(2.36–3.49)	< 0.001	2.26(1.83–2.79)	< 0.001
Sarcopenic obesity	2.93(2.33–3.69)	< 0.001	1.82(1.38–2.40)	< 0.001	6.90(4.93–9.67)	< 0.001	3.99(2.50–6.09)	< 0.001

* Multivariable-adjusted for age, sex, living place, education level, smoking, drinking, body mass index, blood glucose, systolic blood pressure, antihypertensive medication, antidiabetic medication and medical history (dyslipidemia, diabetes, cancer, chronic lung disease, kidney disease, liver disease, arthritis, digestive disease, asthma)

### Sensitivity analyses and subgroup analyses

In the sensitivity analysis, the individuals with severe sarcopenia were merged into the sarcopenia group. The association of sarcopenia and sarcopenic overweight with were consistent to the main analyses (Table S1). Moreover, both in the sex and age subgroup analyses, the results showed a significant relationship between higner ADL disability trajectories and sarcopenia, as well as sarcopenia (Table S2 and S3).

## Discussion

In this nationwide longitudinal study of middle-aged and older adults in China, a total of 9113 people (4228 men and 4885 women) were included. We identified three distinct trajectories of ADL disability: a low-low trajectory, a low-mild trajectory and a mild-high trajectory. Our study is the first to analyze the association between long-term changes in ADL disability and sarcopenia. The study found that sarcopenia was associated with persistently higher ADL disability trajectories. Besides, participants with SO exhibited a significantly higher risk of being ADL disabled. Therefore, timely recognition and management of sarcopenia are of great public health significance.

In this present study, three trajectories of ADL disability were identified using the LCTM to explore the temporal patterns of ADL disability. The findings revealed that individuals in the mild-high trajectory group had a higher ADL score, and may need more health care during the life course. Additionally, individuals in the mild-high trajectory group tended to be female, be older, be living in the rural areas and have lower educational levels. Notably, this study showed that sarcopenia is independently associated with ADL disability, which is consistent with the findings of previous reports [29, 30]. A cross-sectional study in Chinese community-dwelling older individuals concluded an association between sarcopenia and disability and poor physical function [19]. Similarly, a four-year prospective study in Brazil suggested that according to the European working group on sarcopenia in older people (EWGSOP) definition, sarcopenia is a risk factor for disability in the older population [31]. A retrospective and longitudinal follow-up study in Turkey suggested that the probable sarcopenia was independently associated with impairment in ADL, but the results may be affected by the use of different thresholds for probability [32].These findings suggested that older adults with sarcopenia should be identified early and provided with targeted intervention to prevent adverse health outcomes [33].

Additionally, we also observed a significant relationship between sarcopenia obesity and ADL disability trajectories. It suggested that obesity may aggravate the negative impact of sarcopenia on ADL disabilities. Several previous studies reported that the core biological factors related to SO are age-related changes in body composition and metabolism, which may cause functional impairment and physical illnesses [15, 34, 35]. However, several previous studies of the SO yielded contradicting conclusions [36–38]. It is noteworthy that further research based on larger samples is needed to increase the basic knowledge in this area. In the subgroup analyses of gender and age, there was a significant interaction between sarcopenia and changes in ADL disabilities, similar to the results of the main analyses. However, this relationship was not significant between ADL disabilities and sarcopenia alone. It suggested that obesity appear to be involved in the relationship between sarcopenia and ADL disabilities [39]. Therefore, more research is needed to investigate the potential mechanism by which obesity interacts with this relationship.

This study was conducted based on a nationally longitudinal survey with a long follow-up period in middle-aged and older Chinese adults, and it used the ADL disability trajectories, instead of a single time-point assessment, as outcome measures. This study is the first to analyze the association between sarcopenia and long-term changes in ADL in China. In addition, individuals with possible sarcopenia were analyzed, which provides recommendations for those who do not meet the criteria for sarcopenia diagnosis but still suffer from related symptoms. Furthermore, the subgroups and sensitivity analyses were conducted, which accounted for some confounding factors that might play roles in the association between ADL disabilities and sarcopenia. Nevertheless, the limitations of this study should also be acknowledged in this study. First, the definition of sarcopenia is based on the AWGS 2019 criteria, in which muscle mass is assessed using the ASM anthropometric equation applicable to the Chinese population in CHARLS, rather than using the recommended dual X-ray absorptiometry (DXA) measurement. However, it has been proven that the equation is very consistent with DXA. Second, because the current study was a secondary analysis of prospective data from CHARLS, the participants in this study was restricted to Chinese residents aged 45 years and older. Therefore, there may be selection bias, and the conclusions in this study may not be generally applicable to all sarcopenic population. Due to the missing and uncollected data in the survey, several confounding factors, such as physical activity and socioeconomic status, were not considered in our study. Future studies incorporating more patient characteristics and influencing factors are needed to elucidate the mechanism underlying the relationship between ADL disabilities and sarcopenia. In conclusion, the present study showed that sarcopenia was associated with ADL disability over time and obesity may influence the relationship between sarcopenia and ADL disability among Chinese middle-aged and older adults. Thus, sarcopenia prevention and treatment can be used as a therapeutic measure to reduce ADL disability in older individuals. Future studies should pay more attention to clarify the mechanisms of obesity on sarcopenia in older population.

## Conclusions

Among middle-aged and older Chinese adults, the current study identified three distinct trajectories of ADL disability, and individuals with sarcopenia were more likely to be in higher trajectory groups. Besides, SO was associated with higher risk of ADL disability. Further interventions aimed at preventing ADL disability should be targeted at older adults with sarcopenia and SO.

### Electronic supplementary material

Below is the link to the electronic supplementary material.

Supplementary Material 1

## Data Availability

No datasets were generated or analysed during the current study.
