# Supplementary material for: Associations between Sarcopenia and trajectories of activities of daily living disability: a nationwide longitudinal study of middle-aged and older adults in China from 2011 to 2018
Source: Arch Public Health. 2024 Jun 25;82:97. doi: 10.1186/s13690-024-01329-x (PMC11197329; doi:10.1186/s13690-024-01329-x)
Supplement: Supplementary file 1 — Supplementary Material 1 [file 13690_2024_1329_MOESM1_ESM.docx]

**Table S1. Sensitivity analysis the association between sarcopenia status and ADL trajectories among the participants of CHARLS in China from 2011 through 2018.**

| Characteristics | Low-mild V.S. Low-low | | | | Mild-high V.S. Low-low | | | |
| --- | --- | --- | --- | --- | --- | --- | --- | --- |
|  | Crude model  OR (95% CI) | *P* | Adjusted model  OR (95% CI) | *P* | Crude model  OR (95% CI) | *P* | Adjusted model  OR (95% CI) | *P* |
| **Sarcopenia status^*^** | | | | | | | | |
| No sarcopenia | 1.00 (ref) |  | 1.00 (ref) |  | 1.00 (ref) |  | 1.00 (ref) |  |
| Possible sarcopenia | 1.95(1.75-2.17) | <0.001 | 1.53(1.36-1.72) | <0.001 | 3.95(3.26-4.79) | <0.001 | 2.36(1.89-2.95) | <0.001 |
| Sarcopenia | 2.55(2.22-2.04) | <0.001 | 1.51(1.28-1.79) | <0.001 | 6.22(4.98-7.75) | <0.001 | 2.66(2.00-3.53) | <0.001 |
| **Sarcopenic overweight status^#^** | |  |  |  |  |  |  |  |
| Control | 1.00 (ref) |  | 1.00 (ref) |  | 1.00 (ref) |  | 1.00 (ref) |  |
| Sarcopenia only | 1.06(0.95-1.19) | 0.283 | 1.03(0.87-1.21) | 0.739 | 1.11(0.85-1.44) | 0.454 | 0.99(0.69-1.40) | 0.939 |
| Overweight only | 2.08(1.85-2.35) | <0.001 | 1.47(1.29-1.67) | <0.001 | 4.46(3.56-5.59) | <0.001 | 2.36(1.83-3.05) | <0.001 |
| Sarcopenic overweight | 2.38(2.07-2.74) | <0.001 | 1.67(1.38-2.01) | <0.001 | 5.51(4.31-7.03) | <0.001 | 2.57(1.81-3.64) | <0.001 |

*Those with severe sarcopenia were divided into sarcopenia.

Multivariable-adjusted for age, sex, living place, education level, smoking, drinking, body mass index, blood glucose, systolic blood pressure, antihypertensive medication, antidiabetic medication and medical history (dyslipidemia, diabetes, cancer, chronic lung disease, kidney disease, liver disease, arthritis, digestive disease, asthma).

**Table S2. Association between sarcopenia, sarcopenic obesity status and ADL trajectories according to sex among the participants of CHARLS in China from 2011 through 2018.**

| Characteristics | Low-mild V.S. Low-low | | | | Mild-high V.S. Low-low | | | |
| --- | --- | --- | --- | --- | --- | --- | --- | --- |
|  | Male | *P* | Female | *P* | Male | *P* | Female | *P* |
| **Sarcopenia status^*^** | | | | | | | | |
| No sarcopenia | 1.00 (ref) |  | 1.00 (ref) |  | 1.00 (ref) |  | 1.00 (ref) |  |
| Possible sarcopenia | 1.79(1.51-2.14) | <0.001 | 1.35(1.15-1.584) | <0.001 | 2.80(1.98-3.96) | <0.001 | 2.14(1.60-2.87) | <0.001 |
| Sarcopenia | 1.55(1.18-2.05) | 0.002 | 1.50(1.11-1.85) | <0.001 | 2.37(1.43-3.91) | <0.001 | 2.81(1.99-3.99) | <0.001 |
| **Sarcopenic obesity status^*^** | |  |  |  |  |  |  |  |
| Control | 1.00 (ref) |  | 1.00 (ref) |  | 1.00 (ref) |  | 1.00 (ref) |  |
| Sarcopenia only | 1.14(0.78-1.66) | 0.498 | 1.10(0.82-1.46) | 0.526 | 0.46(0.14-1.53) | 0.204 | 1.23(0.68-2.22) | 0.493 |
| Obesity only | 1.72(1.46-2.03) | <0.001 | 1.37(1.19-1.58) | <0.001 | 2.31(1.66-3.23) | <0.001 | 2.26(1.72-2.98) | <0.001 |
| Sarcopenic obesity | 2.06(1.20-3.54) | 0.008 | 1.71(1.23-2.36) | 0.001 | 5.84(2.54-13.45) | <0.001 | 3.49(2.04-5.96) | <0.001 |

^*^Those with severe sarcopenia were divided into sarcopenia.

Multivariable-adjusted for age, sex, living place, education level, smoking, drinking, body mass index, blood glucose, systolic blood pressure, antihypertensive medication, antidiabetic medication and medical history (dyslipidemia, diabetes, cancer, chronic lung disease, kidney disease, liver disease, arthritis, digestive disease, asthma).

**Table S3. Association between sarcopenia, sarcopenic obesity status and ADL trajectories according to age among the participants of CHARLS in China from 2011 through 2018.**

| Characteristics | Low-mild V.S. Low-low | | | | Mild-high V.S. Low-low | | | |
| --- | --- | --- | --- | --- | --- | --- | --- | --- |
|  | Age<60 | *P* | Age≥60 | *P* | Age<60 | *P* | Age≥60 | *P* |
| **Sarcopenia status** | | | | | | | | |
| No sarcopenia | 1.00 (ref) |  | 1.00 (ref) |  | 1.00 (ref) |  | 1.00 (ref) |  |
| Possible sarcopenia | 1.58(1.36-1.84) | <0.001 | 1.49(1.24-1.78) | <0.001 | 2.67(1.91-3.72) | <0.001 | 2.19(1.61-2.96) | <0.001 |
| Sarcopenia | 1.48(1.11-1.96) | 0.007 | 1.53(1.24-1.89) | <0.001 | 3.32(1.91-5.78) | <0.001 | 2.36(1.69-3.30) | <0.001 |
| **Sarcopenic obesity status** |  |  |  |  |  |  |  |  |
| Control | 1.00 (ref) |  | 1.00 (ref) |  | 1.00 (ref) |  | 1.00 (ref) |  |
| Sarcopenia only | 1.11(0.84-1.47) | 0.473 | 1.18(0.80-1.75) | 0.412 | 0.96(0.47-1.96) | 0.907 | 0.99(0.46-2.11) | 0.972 |
| Obesity only | 1.52(1.31-1.77) | <0.001 | 1.51(1.29-1.77) | <0.001 | 2.59(3.65) | <0.001 | 2.07(1.58-2.71) | <0.001 |
| Sarcopenic obesity | 1.97(1.38-2.82) | <0.001 | 1.62(1.05-2.51) | 0.031 | 3.70(1.89-7.26) | <0.001 | 4.02(2.20-7.34) | <0.001 |

Those with severe sarcopenia were divided into sarcopenia.

* Multivariable-adjusted for age, sex, living place, education level, smoking, drinking, body mass index, blood glucose, systolic blood pressure, antihypertensive medication, antidiabetic medication and medical history (dyslipidemia, diabetes, cancer, chronic lung disease, kidney disease, liver disease, arthritis, digestive disease, asthma).
